# Digital dashboards as tools for regional influenza monitoring

**DOI:** 10.5365/wpsar.2017.8.2.003

**Published:** 2017-08-30

**Authors:** Sarah Hamid, Leila Bell, Erica L. Dueger

**Affiliations:** a Division of Health Security and Emergencies, WHO Regional Office for the Western Pacific.

Yearly seasonal epidemics of influenza, an acute viral respiratory disease, pose a substantial health burden on all age groups worldwide. (*1*) In addition, zoonotic influenza viruses circulating in animal populations cause occasional infections in humans. Influenza is a priority disease for regional surveillance in the World Health Organization (WHO) Western Pacific Region, where several zoonotic influenza viruses have infected humans in recent years. (*2*) Effective risk assessment for influenza is supported through the use of multiple sources of surveillance information. However, bringing together different streams of data and information for analysis can be challenging. Online visualization and analytics tools help to synthesize and disseminate various data sources for risk assessment and public health action. Herein we describe digital dashboards built by the WHO Regional Office for the Western Pacific to share regional influenza data.

Surveillance systems for influenza are well established in many high-income countries, allowing for continuous epidemiological and virological characterization of circulating viruses. (*3*) Data from these systems are routinely analysed to monitor trends, assess risk, plan interventions and allocate resources. (*4*) In recent years, many lower- and middle-income countries have established syndromic surveillance systems for influenza that are also generating repositories of epidemiological and virological data. (*5*)

WHO’s Global Influenza Surveillance and Response System (GISRS) collects and collates data on circulating strains of influenza viruses to inform vaccine composition recommendations, conduct risk assessments and monitor antiviral susceptibility. (*6*) In the Western Pacific Region, GISRS includes 21 National Influenza Centres (NICs) in 15 countries and areas that receive respiratory specimens from a range of sources in their respective countries. NICs upload virologic data to FluNET, a publicly available web-based tool for influenza virological surveillance launched by WHO in 1997. Recently, WHO established FluMART, a global data-sharing platform that links national influenza epidemiological data with virological FluNET data in a single global database. Further efforts are needed to analyse and disseminate these routinely collected data to inform policy and stimulate public health action.

**Fig. 1:**
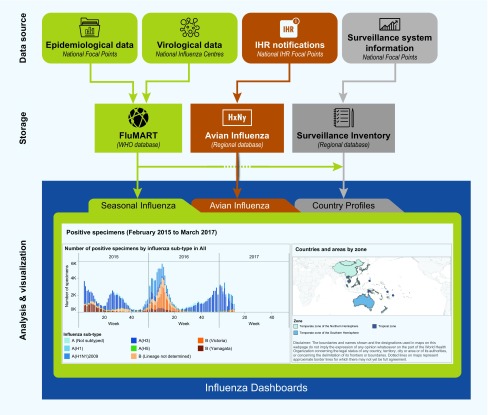
Schematic diagram of data sources and flow for regional influenza dashboards

Interactive, web-based surveillance reporting platforms allow users to access timely disease information collected by national surveillance systems and view these data in a dynamic manner to meet their particular needs. (*7*) Several agencies, including various WHO offices and the United States Centers for Disease Control and Prevention, have developed platforms to disseminate influenza surveillance information. (*8*-*10*) To enhance regional information-sharing and the use of multiple sources of information for risk assessment under the Asia Pacific Strategy for Emerging Diseases and Public Health Emergencies, (*11*) and to further global collaboration under the Pandemic Influenza Preparedness Framework Partnership Contribution Implementation Plan, the WHO Regional Office for the Western Pacific has developed a set of online interactive influenza dashboards. (*12*) The dashboards summarize overall influenza activity and surveillance capacity in the Region by bringing together laboratory and epidemiological data; national surveillance system information; and data on human infections with avian influenza viruses A(H5N1), A(H5N6) and A(H7N9) notified under the International Health Regulations (IHR) (**Fig. 1**). The site is publicly accessible, allowing communication with various audiences at the national, regional and global levels. Disseminating these data supports risk assessments, thereby narrowing the gap between surveillance and public health action.

This figure shows the four sources and contributors of data to the regional influenza dashboards, where these data are stored and on which dashboards they are displayed. National epidemiological and virological data are stored in a global WHO database (FluMART) and displayed on the Seasonal Influenza and Country Profiles dashboards. Human infections with novel influenza viruses are reported by Member States under the IHR, stored in a regional WHO database and displayed on the Avian Influenza dashboards. National surveillance system information reported by individual Member States through a questionnaire is stored in a regional database and displayed on the Country Profiles dashboards.

To develop the dashboards, a proof of concept prototype using simulated data and designed with Tableau 9.0 (Tableau Software, Seattle, Washington) was shared for feedback with potential stakeholders through an online questionnaire and discussions at a regional influenza forum in August 2015. Revisions were made accordingly and a single data request was sent to countries to gather national surveillance system information and epidemiological data for inclusion in WHO databases. This collaborative effort resulted in a pilot site that was presented to key stakeholders in 2016, before launching publicly.

The regional influenza dashboards include individual dashboards that display seasonal and avian influenza data through interactive maps, graphs and tables (**Fig. 1**). Surveillance system information is currently provided for 35 countries and areas, virological data for 15 countries and areas and epidemiological data for 28 countries and areas. Basic epidemiological data on human infections with avian influenza A(H5N1), A(H5N6) and A(H7N9) viruses are also displayed. The platform facilitates creation of graphics that can be tailored to meet specific needs and downloaded directly. For example, users can compare measures of seasonal influenza activity, including counts of influenza-like illness or severe acute respiratory infection cases, number of deaths and number of positive virological specimens between different time periods within the same country or between different countries within the Region. Additionally, users can map human infections with avian influenza viruses at the provincial level for specified time periods to visualize the spread of infections over time. Links directing users to national surveillance information and an archive of biweekly regional reports are also available.

Still in the early stages of production, the dashboards have some limitations. Tableau software is compatible with newer versions of web browsers so computers running older versions, such as Internet Explorer 8, 9 and 10, may not display the dashboards. Due to differences between countries in reporting frequency, surveillance methodology, case definitions and continuity of reporting by individual surveillance sites caution must be taken when comparing across years or between countries and areas. Sustainability is also a concern in the adoption of new and innovative technologies. However, steps to address this concern have been taken and include the efficient leveraging of existing global and regional databases that link to the dashboards without duplicate data entry by WHO Member States.

The Western Pacific regional influenza dashboards will facilitate the use of a growing amount of virological and epidemiological surveillance data as a basis for public health action. Not only do they present regional data, but they also support countries with limited capacity to maintain national reporting platforms. By linking different information sources, they support a regional system that can serve as an operational hub to inform risk assessment and decision-making. In the face of a pandemic, regional dashboards could provide both baseline and real-time surveillance information for risk assessment. Moving forward, it will be useful to incorporate animal sector data and information from vaccination surveys such as the WHO-UNICEF Joint Reporting Form to bring together disparate information sources in a single platform for better informed regional risk assessments. Regional monitoring and assessment of other priority diseases could be enhanced by developing similar dashboards. In the future, linkages between focused disease-specific platforms could yield comprehensive overviews of national and regional public health.
